# Age-Dependent Assessment of Genes Involved in Cellular Senescence, Telomere, and Mitochondrial Pathways in Human Lung Tissue of Smokers, COPD, and IPF: Associations With SARS-CoV-2 COVID-19 ACE2-TMPRSS2-Furin-DPP4 Axis

**DOI:** 10.3389/fphar.2020.584637

**Published:** 2020-09-09

**Authors:** Krishna P. Maremanda, Isaac K. Sundar, Dongmei Li, Irfan Rahman

**Affiliations:** ^1^Department of Environmental Medicine, University of Rochester Medical Center, Rochester, NY, United States; ^2^Department of Clinical and Translational Research, University of Rochester Medical Center, Rochester, NY, United States

**Keywords:** mitochondria, cellular senescence, telomere, DNA damage, aging, smokers, idiopathic pulmonary fibrosis, chronic obstructive pulmonary diseases

## Abstract

**Background:**

Aging is one of the key contributing factors for chronic obstructive pulmonary diseases (COPD) and other chronic inflammatory lung diseases. Here, we determined how aging contributes to the altered gene expression related to mitochondrial function, cellular senescence, and telomeric length processes that play an important role in the progression of COPD and idiopathic pulmonary fibrosis (IPF).

**Methods:**

Total RNA from the human lung tissues of non-smokers, smokers, and patients with COPD and IPF were processed and analyzed using a Nanostring platform based on their ages (younger: <55 years and older: >55 years).

**Results:**

Several genes were differentially expressed in younger and older smokers, and patients with COPD and IPF compared to non-smokers which were part of the mitochondrial biogenesis/function (*HSPD1*, *FEN1*, *COX18*, *COX10*, *UCP2 & 3*), cellular senescence (*PCNA*, *PTEN*, *KLOTHO*, *CDKN1C*, *TNKS2*, *NFATC1 & 2*, *GADD45A*), and telomere replication/maintenance (*PARP1*, *SIRT6*, *NBN*, *TERT*, *RAD17*, *SLX4*, *HAT1*) target genes. Interestingly, *NOX4* and *TNKS2* were increased in the young IPF as compared to the young COPD patients. Genes in the mitochondrial dynamics and quality control mechanisms like *FIS1* and *RHOT2* were decreased in young IPF compared to their age matched COPD subjects. ERCC1 and *GADD45B* were higher in young COPD as compared to IPF. Aging plays an important role in various infectious diseases including the SARS-CoV-2 infection. Lung immunoblot analysis of smokers, COPD and IPF subjects revealed increased abundance of proteases and receptor/spike protein like TMPRSS2, furin, and DPP4 in association with a slight increase in severe acute respiratory syndrome coronavirus 2 (SARS-CoV-2) receptor ACE2 levels.

**Conclusions:**

Overall, these findings suggest that altered transcription of target genes that regulate mitochondrial function, cellular senescence, and telomere attrition in the pathobiology of lung aging in COPD and IPF is associated with alterations in SARS-CoV-2 ACE2-TMPRSS2-Furin-DPP4 axis as pharmacological targets for COVID-19.

## Introduction

Aging is an important factor influencing the overall lung health and function (Wahba, 1983; Skloot, 2017). Lung function declines with age after lung maturation. Evidences suggest that a significant contribution of various environmental factors influence the aging lung (Rojas et al., 2015). According to the Behavioral Risk Factor Surveillance System (BRFSS) data from 2017, 6.2% (age-adjusted) US adults were reported to have chronic obstructive pulmonary disease (COPD) (Wheaton et al., 2019). Further, there has been an increasing reports of young COPD population visiting for different hospital services (Gershon et al. 2019). Aging influences many chronic lung diseases, such as COPD, idiopathic pulmonary fibrosis (IPF), and asthma. Also chronic lung diseases like asthma and IPF share some of the common yet distinct features compared to COPD (Maghsoudloo et al., 2020). Environmental stress factors like smoking remains a common influencing factor for the disease progression in all these three cases.

Cigarette smoke (CS) is one of the strongest contributing risk factors in the pathogenesis of COPD along with the decline in lung function (Rahman and Adcock, 2006). Cellular senescence is a process of irreversible cell cycle arrest, having both beneficial and harmful effects depending on the cell state (Birch et al., 2018). CS plays a role in advancing the lung aging by altering the process of cellular senescence (Nyunoya et al., 2006). Several factors including oxidative stress influence the process of cellular senescence. Telomeres and mitochondria play a major role in influencing the process of cellular senescence and are often associated with maintaining the lung cellular health (Liu et al., 2002; Passos and von Zglinicki, 2005; Birch et al., 2018). Earlier reports from our laboratory and others have shown that smoking and COPD is associated with the mitochondrial damage and dysfunction, altering the process of cellular metabolism and function (Ahmad et al., 2015; Lerner et al., 2016). Similarly, telomere dysfunction was also associated with smoking and COPD (Morla et al., 2006; de-Torres et al., 2017). Mitochondrial and telomere dysfunction also play a causative role in the progression of IPF (Povedano et al., 2015; Molina-Molina and Borie, 2018; Zank et al., 2018).

Several molecular mechanisms were identified in relation to CS causing COPD pathogenesis and associated complications (Putcha et al., 2015). CS alters several key cellular functions, among them the crucial genes related to mitochondrial function, cellular senescence, and telomeric length were selected in the current study to observe for any differential changes among young and old age groups categorized as non-smokers, smokers, and COPD groups. Our previous studies showed independent contributions of these canonical signaling pathways and how they contribute toward the development of premature lung aging in chronic lung diseases, such as COPD/emphysema (Yao et al., 2012; Yao and Rahman, 2012; Ahmad et al., 2017; Rashid et al., 2018). Accumulating evidence suggest the close relationship of all these three pathways in influencing lung aging and disease (Lee et al., 1998; Saretzki et al., 2003; Passos and von Zglinicki, 2005). Senescent cells are found in many age-related/chronic diseases (Tchkonia and Kirkland, 2018). Studies from our laboratory showed that mice from different age group when exposed to chronic air and CS influence the process of lung inflammation and senescence. Chronic CS exposure in lung epithelial cells and mice increases several markers of cellular senescence (Nyunoya et al., 2006; Fujii et al., 2012). Recently, it was reported that serum from COPD patients can induce senescence in lung epithelial cells (Kuznar-Kaminska et al., 2018), giving strength to the importance of this area that needs to be explored further. Several DNA damaging agents present in smoke, which may activate the DNA damage response, thereby influencing telomere function leading to accumulation of senesced cells. However, recent meta-analysis suggests that even though smokers are associated with shorter telomere length, the study implicates that smoking does not accelerate the telomere attrition in leucocytes (Bateson et al., 2019).

Smoking and COPD conditions altered the expressions of many key genes involved in the above three crucial pathways of cellular maintenance. The current study was undertaken to determine the changes in genes related to mitochondrial biogenesis and function, telomere function, and cellular senescence with respect to their age in human lungs. This study is important in unraveling some of the potential biomarkers to differentiate and follow the course of aging in COPD. Keeping in view the importance of these genes in both COPD and IPF, we have also made comparisons between the similar age grouped COPD and IPF subjects, using the same set of gene panels.

Aging is one of the key components, which decides the subject’s susceptibility to various diseases and infections presumably due to inflammaging (Gardner, 1980). The recent pandemic of severe acute respiratory syndrome coronavirus 2 (SARS-CoV-2) was thought to affect more elderly people especially men (Bonafe et al., 2020; Koff and Williams, 2020; Sargiacomo et al., 2020). Men with age-related comorbidities have a higher coronavirus disease 2019 (COVID-19) mortality rate (Zhao et al., 2020). Comorbidities like cardiovascular disease, diabetes, and chronic respiratory diseases present a high mortality rate (Emami et al., 2020). Studies involving the role of lung aging and senescence play an important role in understanding the role of the multiple players involved in combating SARS-CoV-2 infection and can discover potential druggable targets. Considering the important role played by some of the crucial receptors and targets in COVID-19 (Hoffmann et al., 2020), we determined the protein expression of SARS-CoV-2 receptor angiotensin converting enzyme 2 (ACE2) and aiding proteases like transmembrane protease serine protease-2 (TMPRSS-2) and spike protein convertase furin in lung homogenates of non-smokers, smokers, COPD, and IPF subjects. We also examined the levels of dipeptidyl peptidase 4 (DPP4), the receptor for the Middle East respiratory syndrome coronavirus, (MERS-CoV) (Seys et al., 2018).

## Methods

### Scientific Rigor and Reproducibility

Rigorous and unbiased approaches were used to ensure full and detailed reporting of both methods and analyzed data.

### Ethical Approval: Institutional Biosafety and Review Board Approvals

#### Ethics Statement

The current study was approved for the procurement of the human lung tissues as de-identified tissues by the Materials Transfer Agreement and Procurement (Institutional Review Board, IRB), and laboratory protocols by the Institutional Biosafety Committee, IBC of the University of Rochester Medical Center, Rochester, NY, with Project Code: DRAI1 001 Protocol: 004, Date of approval and IRB/IBC approvals 2/11/2017 and 2/7/2018, and 9/29/2017 and 2/10/2017, University material transfer agreement (MTA) signed on the above dates as well. Patients’ data or patients are not directly involved in this study as the lung tissues were procured from several agencies (see below). All patients/subjects were of age 21 and above. All methods were carried out in accordance with relevant guidelines and regulations of the University of Rochester, Rochester, NY.

### Human Lung Tissues

The human peripheral lung tissues from non-smokers, smokers, and COPD/IPF were procured/obtained from the NDRI (National Disease Research Interchange; the samples were collected from patients with various cause of deaths reported such as cardiac arrest or trauma/accidents, for most of the samples lower peripheral lung lobes were used or as supplied), LTRC (Lung Tissue Research Consortium of the National Heart Lung Blood Institute, NHLBI), and Department of Medicine and Pathology, and of the University of Helsinki Hospital, Finland as described in our previous reports (Sundar et al., 2017). The clinical characteristics of the subjects used in the current study are given in **Table 1**. The subjects were broadly classified into two age groups: young age (≤55 years) group, old age (>55 years) group, as per previous studies (Sanchez-Salcedo et al., 2014). Although there were some co-morbid conditions reported for the specimens from COPD patients (which were on various medications), the tissues were assigned to different groups based on the age, smoking status (current/ex-smokers), and lung disease status (normal vs. smoker’s, normal vs. COPD) reported during the procurements of the specimens. As described in the above samples, the following samples were obtained in the same way for further disease-wise comparison. Additional comparisons were made between COPD (8 additional samples from above groups were added in addition to the 24 samples mentioned in **Table 1**) and IPF lung samples (3 in young IPF, 13 in old IPF) based on their age to determine for the changes in the same custom gene panel (**Supplementary Table 1**).

**Table 1:** Clinical characteristics of non-smokers, smokers, and COPD and IPF subjects/patients.

Groups	Young Non-smokers	Young Smokers	Young COPD	Young IPF	Old Non-smokers	Old Smokers	Old COPD	Old IPF
Sex/Gender ratio	4:2 (M:F)	3:3 (M:F)	4:1 (M:F)	3:0 (M:F)	3:1 (M:F)	2:2 (M:F)	1:6 (M:F)	10:3 (M:F)
Sex/Gender (Percentage)	66.67%/33.33%	50%/50%	80%/20%	100%/0	75%/25%	50%/50%	14.28%/85.72%	76.92%/23.08%
Average Age (range in years)	38.17 (25–55)	41 (26–54)	49.2 (45–53)	54.33 (54–55)	68.5 (63–78)	67.75 (61–81)	70 (65–73)	69.30 (56–79)
Smoking history (Pack years)	N/A	16.83 (1–30)	38.33 (30–45)	N/A	N/A	66.25 (40–120)	41.67 (15–58)	N/A

### RNA Isolation From Lung Tissues

Total RNA was extracted from the human lung tissues stored at −80°C or in RNAlater, using Direct-Zol RNA miniprep plus kit (Zymo research, R2071) according to the manufacturer’s instructions. RNA concentration was measured using a Nanodrop 1000 (Thermo Fisher Scientific, USA). Various genes involved in mitochondrial biogenesis and function, telomere replication and maintenance, and cellular senescence pathways were included in the custom code sets (**Supplementary Table 2**). The code set contained a total of 112 genes including 6 reference genes (*Abcf1*, *Hprt*, *Polr1b*, *Rplp0*, *Ldha*, *Gusb*) for gene normalization (**Supplementary Table 3**). The code sets were identified by overlapping the existing panels of the above cellular pathways and the distinct genes were used as described in the code sets. The samples were sent for analysis and processed through the NanoString nCounter system (NanoString Technologies Seattle, WA, USA). A total of 400-ng RNA was submitted after adjusting the samples to a minimum of 20 µg/µl as per the requirements.

### Validation of Gene Targets Using Quantitative Real-Time PCR

Selected mRNA (prepared as mentioned above), which were found to be significantly and differentially altered were further validated for their expression using qPCR (three samples per group, run in duplicates) used in the study as described earlier (Maremanda et al., 2019). The primers were obtained from Bio-Rad as described below with catalog numbers along with PCR conditions (Initial denaturation at 95°C 10 min followed by 40 cylces at 95°C 15 s and 60°C 1 min, fluorescence intensity was measured during the end of 60°C incubation, melting curve analysis was performed after this reaction (65°C for 5 s, and then 0.5°C for 5 s until 95°C). Specific primer sets are given with qHsa code number by the manufacturer on their data sheets, such as PrimePCR™ SYBR^®^ Green Assay: KL, Human part #10025636; PrimePCR™ SYBR^®^ Green Assay: HSPD1, Human, qHsaCED0056432; PrimePCR™ SYBR^®^ Green Assay: KL, Human, qHsaCID0011259; PrimePCR™ SYBR^®^ Green Assay: CDKN1C, Human, qHsaCED0044284; PrimePCR™ SYBR^®^ Green Assay: PARP1, Human, qHsaCED0045162; PrimePCR™ SYBR^®^ Green Assay: TERT, Human, qHsaCID0009247; PrimePCR™ SYBR^®^ Green Assay: SIRT6, Human, qHsaCID0014307; PrimePCR™ SYBR^®^ Green Assay: NOX4, Human, qHsaCID0012395; PrimePCR™ SYBR^®^ Green Assay: TNKS2, Human, qHsaCID0015641; PrimePCR™ SYBR^®^ Green Assay: FIS1, Human, qHsaCED0038514; PrimePCR™ SYBR^®^ Green Assay: RHOT2, Human, qHsaCID0011549; PrimePCR™ SYBR^®^ Green Assay: COX18, Human, qHsaCID0017694; PrimePCR™ SYBR^®^ Green Assay: COX10, Human, qHsaCED0041926; PrimePCR™ SYBR^®^ Green Assay: UCP2, Human, qHsaCID0013753; PrimePCR™ SYBR^®^ Green Assay: UCP3, Human, qHsaCED0038190; PrimePCR™ SYBR^®^ Green Assay: PCNA, Human, qHsaCID0012792; PrimePCR™ SYBR^®^ Green Assay: PTEN, Human, qHsaCED0036796; PrimePCR™ SYBR^®^ Green Assay: NFATC1, Human, qHsaCED0044370; PrimePCR™ SYBR^®^ Green Assay: NFATC2, Human, qHsaCID0009393; PrimePCR™ SYBR^®^ Green Assay: GADD45A, Human, qHsaCED0036441; PrimePCR™ SYBR^®^ Green Assay: NBN, Human, qHsaCID0010883; PrimePCR™ SYBR^®^ Green Assay: RAD17, Human, qHsaCED0045586; PrimePCR™ SYBR^®^ Green Assay: SLX4, Human, qHsaCID0011115; PrimePCR™ SYBR^®^ Green Assay: HAT1, Human, qHsaCID0008873; PrimePCR™ SYBR^®^ Green Assay: NOX4, Human, qHsaCID0012395; PrimePCR™ SYBR^®^ Green Assay: ERCC1, Human, qHsaCID0008822; PrimePCR™ SYBR^®^ Green Assay: GADD45B, Human, qHsaCED0002576; PrimePCR™ SYBR^®^ Green; Assay: GAPDH, Human, qHsaCED0038674, and for *FEN1* (F-CACCTGATGGGCATGTTCTAC, R- CTCGCCTGACTTGAGCTGT) and *18S* (F- GTAACCCGTTGAACCCCATT, R- CCATCCAATCGGTAGTAGCG), used as internal control were obtained from integrated DNA Technologies. Bio-Rad CFX96 real-time system was used to run the experiments in a 96 well plate format. Each 10-μl reaction mixture consist of RT SYBR green PCR master mix, ddH_2_O, primer pairs, and cDNA template. Relative RNA abundance was quantified by the comparative 2^–ddCt^ method. Results were represented as pairwise comparisons to compliment the observations made by NanoString analysis. Student t-test was used to determine the level of significance between two groups, while ANOVA was used for multiple group comparisons.

### Western Blot Analysis in Human Lung Homogenates

Total protein assayed by bicinchoninic acid (BCA) method were isolated from the lung homogenates of non-smokers, smokers, COPD, and IPF in RIPA buffer with protease inhibitors, which were reduced and separated using the pre-made polyacrylamide gels (Bio-Rad) (Maremanda et al., 2019). The transferred membranes were probed for some of the important protein involved in the COVID-19, like TMPRSS2 (ab92323), furin (ab183495), DPP4 (ab28340), and ACE2 (ab108252). All the antibodies used in the current study were procured from the Abcam and were used at 1:1000 dilution in blocking buffer. The relative expression and equal loading as assessed using the Ponceau S staining or β-actin after stripping of the blots. Imaging was done using Bio-Rad Chemidoc and blots were analysed using image lab densitometry.

### Data Processing and Statistical Analysis

Nanostring mRNA counts were first normalized using the *NanoStringNorm* function in the statistical analysis software R (version 3.6.1) on log2 transformed data. Geometric mean of reference genes was used to remove the technical variation and background expression. Differential analysis was conducted using linear models in the limma (version 3.44.3) package (R/Bioconductor) after adjusting for the gender difference. Comparison among different experimental groups were performed using linear contrasts within the linear model framework; moderated *t* statistics was used to determine the differences in the gene expression levels between groups with empirical Bayes approach. The Benjamini-Hochberg procedure was used to adjust the *p* values to control the false discovery rates at 5%. The analyzed data was represented in the graphs as y-axis showing the negative log10 P-value and x-axis representing log2 fold change across each pairwise comparisons as described previously (Sundar et al., 2018). The significantly altered gene data were shown as dot plot representation. Four random samples from each age group were used for comparisons among non-smokers, smokers, and COPD groups (as given in **Supplementary Table 2**). Comparisons with IPF includes all the samples as mentioned in the **Table 1** and **Supplementary Table 3**.

## Results

Overall, the study consisted of 24 lung tissue samples from different sources as mentioned above. The collected tissues were classified into six different groups based on age, the smoking and disease status. Further, comparative gene analysis was also done based on the smoking and disease status irrespective of the age. There were no genes in common that were changed in any of the comparisons involving all the three groups, i.e., non-smokers, smokers, and COPD.

### Differentially Expressed Genes in Young Non-Smokers Versus Young Smokers Versus Young COPD Groups

**Figure 1:**
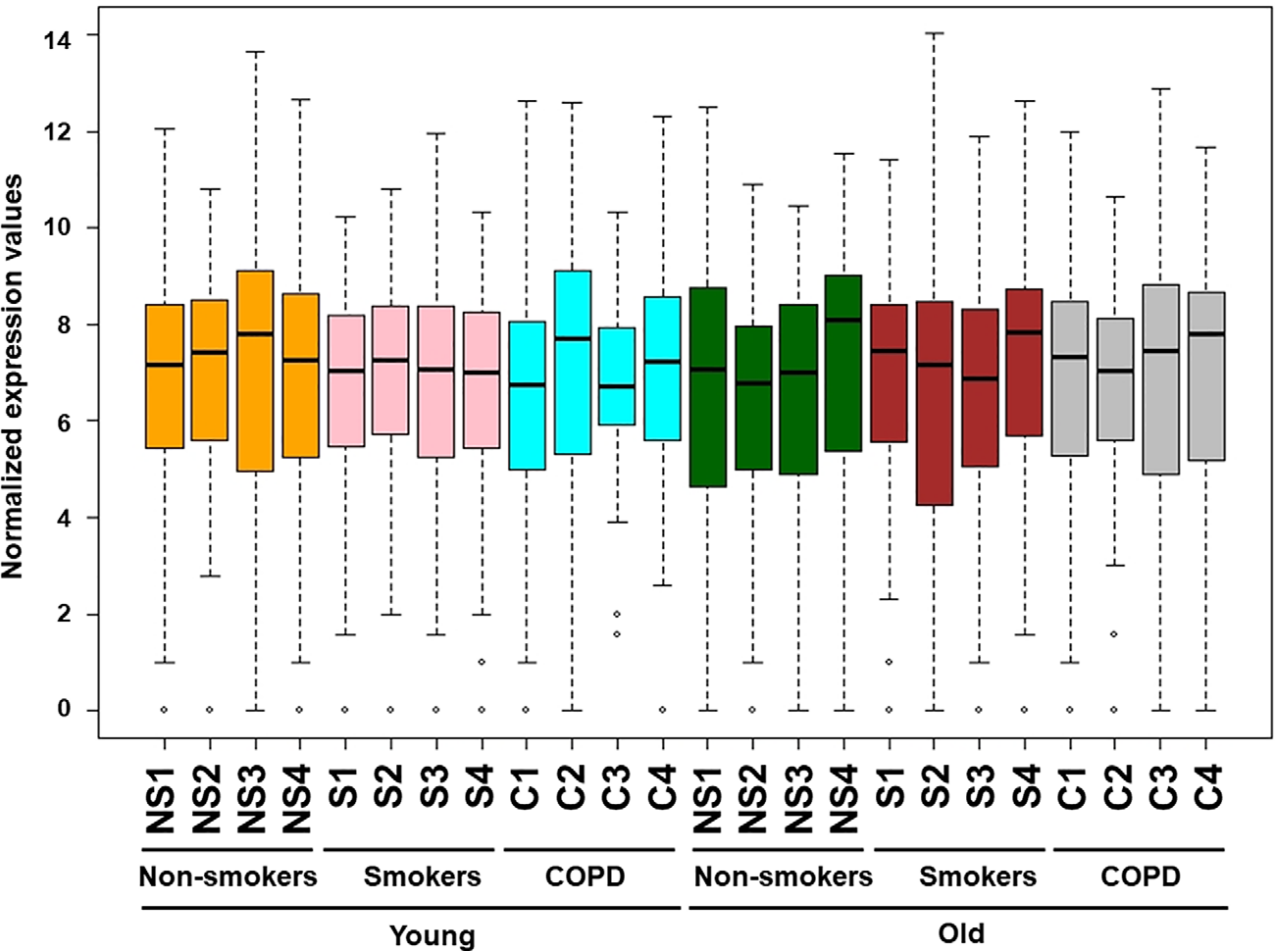
Boxplot analysis of normalized mRNA transcript analyzed by NanoString. Boxplot shows distribution of normalized gene expression levels from young and old non-smokers, smokers, and COPD subjects.

**Figure 2:**
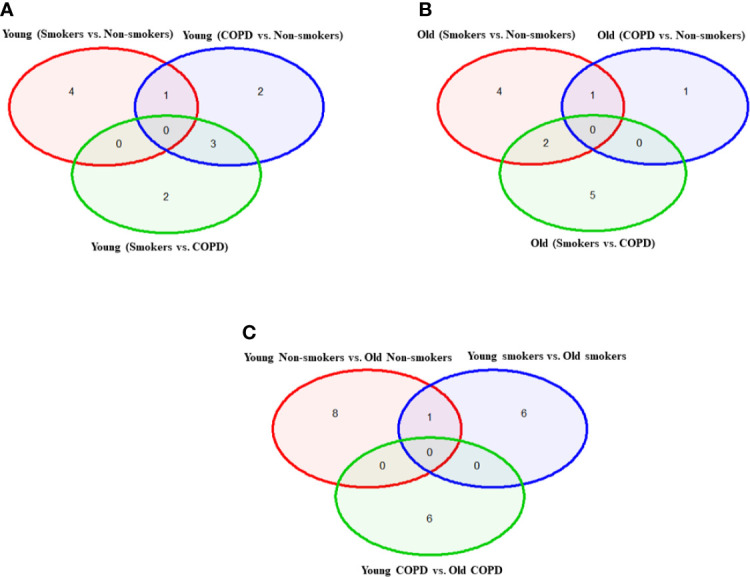
Venn diagram showing the number of altered mRNA transcripts analyzed by NanoString. Venn diagram representing the number of gene changes among non-smokers, smokers, and COPD, which were further divided for pairwise comparisons into **(A)** young vs. young, **(B)** old vs. old, and **(C)** young vs. old aged subjects. Lung RNA was isolated, processed, and analyzed by NanoString. Normalized gene expressions were used for all the comparisons.

**Figure 3:**
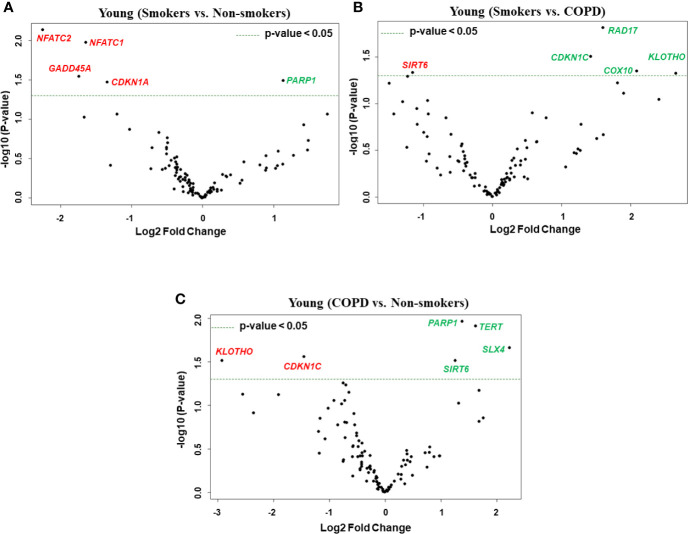
Volcano plots showing differentially expressed genes related to mitochondrial biogenesis and function, telomere replication and cellular senescence genes among non-smokers, smokers, and COPD subjects. Altered genes in comparisons between **(A)** young (smokers vs. non-smokers), **(B)** young (smokers vs. COPD), and **(C)** young (COPD vs. non-smokers). The genes differentially expressed in each comparison are indicated in green (increased) and red (decreased) colors. The green dotted horizontal line indicates the significance threshold of p-values from comparisons at *P* < 0.05. The Benjamini-Hochberg procedure was further used to adjust the p-values to control the false discovery rate at 5%.

First, we analyzed differentially expressed transcript levels among young non-smokers vs. young smokers, young smokers vs. young COPD and young non-smokers vs. young COPD (**Figure 1**). We found five genes were differentially expressed in young non-smokers vs. young smokers’ pairwise comparison. Out of five genes, the transcript levels of four genes (*NFATC1*, *NFATC2*, *GADD45A*, and *CDKN1A*) were decreased and one gene (*PARP1*) was increased in the young smokers as compared to young non-smokers group (**Figures 2** and **3A**). Next, we compared genes differentially expressed in young smokers vs. young COPD pairwise comparison. Out of five genes, transcript levels of one gene (*SIRT6*) was decreased and the remaining four genes (*RAD17*, *CDKN1C*, *COX10*, and *KLOTHO*) were significantly increased in young smokers as compared to the young COPD group (**Figures 2A** and **3B**). Finally, we found six genes differentially expressed among young non-smokers vs. young COPD group pairwise comparison. Out of six genes, the transcript levels of two genes *CDKN1C* and *KLOTHO* that belong to cellular senescence panel were decreased in the young COPD as compared to young non-smokers group. While the transcript levels of the remaining four genes *PARP1*, *SIRT6*, *TERT*, and *SLX4* were increased in the young COPD as compared to the young non-smokers group (**Figures 2A** and **3C**). Overall, four genes *PARP1*, *SIRT6*, *KLOTHO*, and *CDKN1C* were among the common target genes that were differentially expressed in the young COPD group as compared to young non-smokers and young smokers groups.

### Differentially Expressed Genes in Old Non-Smokers Versus Old Smokers Versus Old COPD Groups

**Figure 4:**
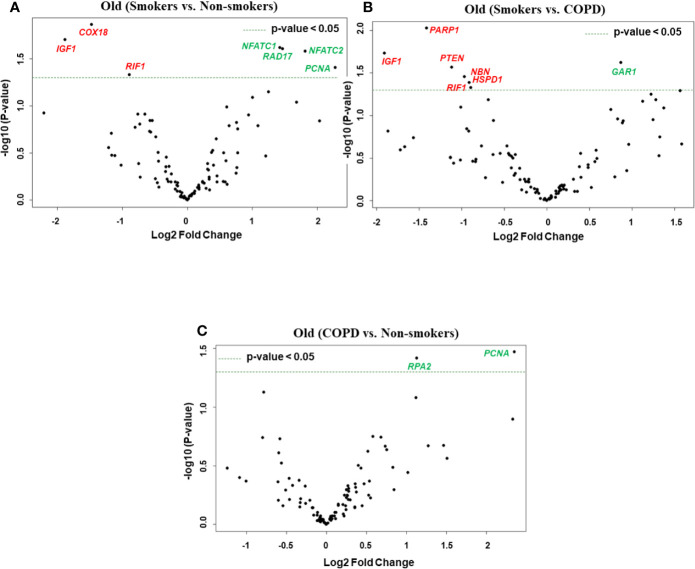
Volcano plots showing differentially expressed genes related to mitochondrial biogenesis and function, telomere replication and cellular senescence genes among non-smokers, smokers, and COPD subjects. Altered genes in comparisons between **(A)** old (smokers vs. non-smokers), **(B)** old (smokers vs. COPD), and **(C)** old (COPD vs. non-smokers). The genes differentially expressed in each comparison are indicated in green (increased) and red (decreased) colors. The green dotted horizontal line indicates the significance threshold of p-values from comparisons at *P* < 0.05. The Benjamini-Hochberg procedure was further used to adjust the p-values to control the false discovery rate at 5%.

Here, we analyzed differentially expressed transcript levels among old non-smokers vs. old smokers, old smokers vs. old COPD, and old non-smokers vs. old COPD groups. Out of seven genes, we found three genes *IGF1*, *COX18*, and *RIF1* were decreased and the remaining four genes *NFATC1*, *NFATC2*, *RAD17*, and *PCNA* were increased in old smokers as compared to old non-smokers group (**Figures 2B** and **4A**). The transcript levels of *IGF1*, *PARP1*, *PTEN*, *NBN*, *HSPD1*, and *RIF1* were decreased and *GAR1* was increased in old smokers as compared to old COPD group (**Figures 2B** and **4B**). Only two genes were affected among old non-smokers and old COPD group; *RPA2* and *PCNA* were increased in old COPD as compared to the old non-smokers group (**Figures 2B** and **4C**). Overall, a total of three genes as mentioned above (*IGF1*, *RIF1*, and *PCNA*) were among the common targets that were found to be differentially expressed in old smokers as compared to old non-smokers and the old COPD groups.

### Altered Gene Expression Levels in Young and Old Non-Smokers Versus Smokers Versus COPD Groups

**Figure 5:**
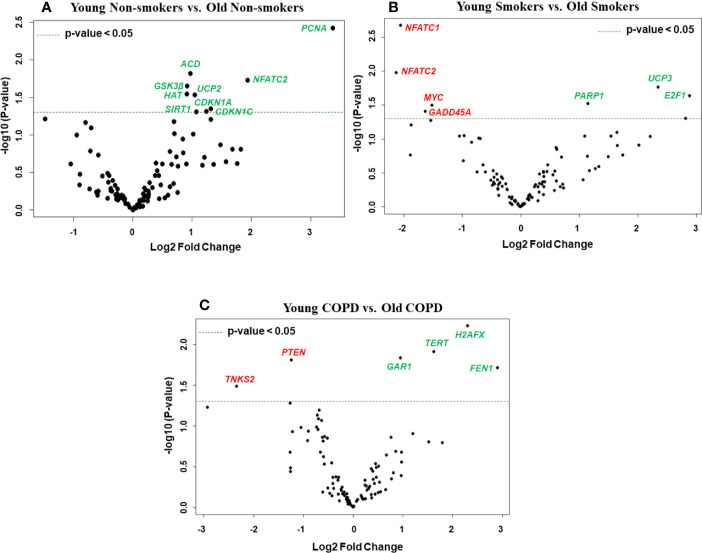
Volcano plots showing differentially expressed genes related to mitochondrial biogenesis and function, telomere replication and cellular senescence genes among non-smokers, smokers, and COPD subjects. Altered genes in comparisons between **(A)** non-smokers (young vs. old), **(B)** smokers (young vs. Old), **(C)** COPD (young vs. old). The genes differentially expressed in each comparison are indicated in green (increased) and red (decreased) colors. The green dotted horizontal line indicates the significance threshold of p-values from comparisons at *P* < 0.05. The Benjamini-Hochberg procedure was further used to adjust the p-values to control the false discovery rate at 5%.

We then analyzed differentially expressed genes among young non-smokers vs. old non-smokers, young smokers vs. old smokers, and young COPD vs. old COPD pairwise comparisons. Transcript levels across different age groups were performed to better understand, whether age influences the measured outcomes in the current study. Accordingly, we found that nine genes were significantly elevated in young non-smokers as compared to old non-smokers group (*PCNA*, *NFATC2*, *ACD*, *GSK3β*, *HAT1*, *UCP2*, *CDKN1A*, *CDKN1C*, and *SIRT1*; **Figures 2C** and **5A**). Although, young smokers show increased transcript levels of *PARP1*, *UCP3*, and *E2F1* genes but decreased levels of *NFATC1*, *NFATC2*, *MYC*, and *GADD45A* as compared to the old smokers group as seen in **Figures 2C** and **5B**. Additionally, two genes (*TNSK2* and *PTEN*) were decreased in the young COPD group as compared to the old COPD group. The transcript levels of *GAR1*, *TERT*, *H2AX*, and *FEN1* tend to increase in the younger COPD group as compared to the old COPD group (**Figures 2C** and **5C**). Interestingly, we noted that transcript levels of *NFATC2* was decreased in lungs of old non-smokers, but increased in lungs of old smokers.

### Combined Analysis of Differentially Expressed Genes Among Non-Smokers, Smokers, and COPD Groups

**Figure 6:**
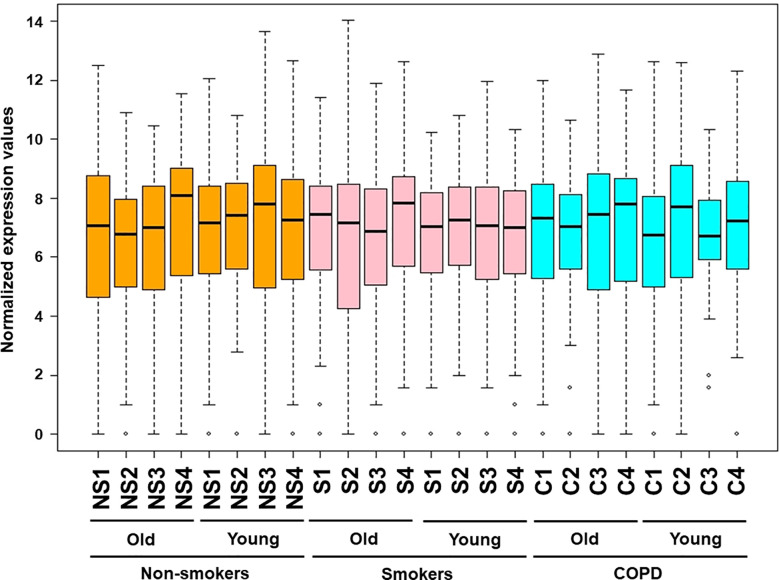
Boxplot analysis of normalized mRNA transcript analyzed by NanoString. Boxplot shows distribution of normalized gene expression levels in combined subjects from non-smokers, smokers, and COPD subjects.

**Figure 7:**
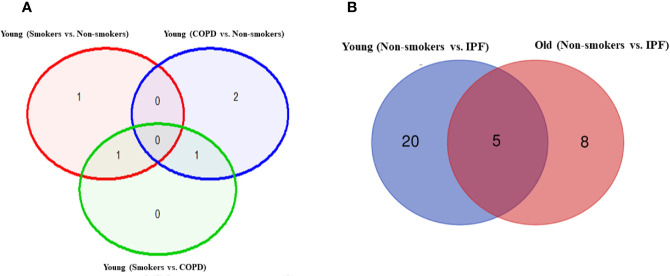
Venn diagram showing the number of altered mRNA transcripts analyzed by NanoString. Venn diagram representing the number of gene changes among **(A)** non-smokers, smokers, and COPD. **(B)** Non-smokers and IPF groups. Lung RNA was isolated, processed, and analyzed by NanoString. Normalized gene expressions were used for all the comparisons.

**Figure 8:**
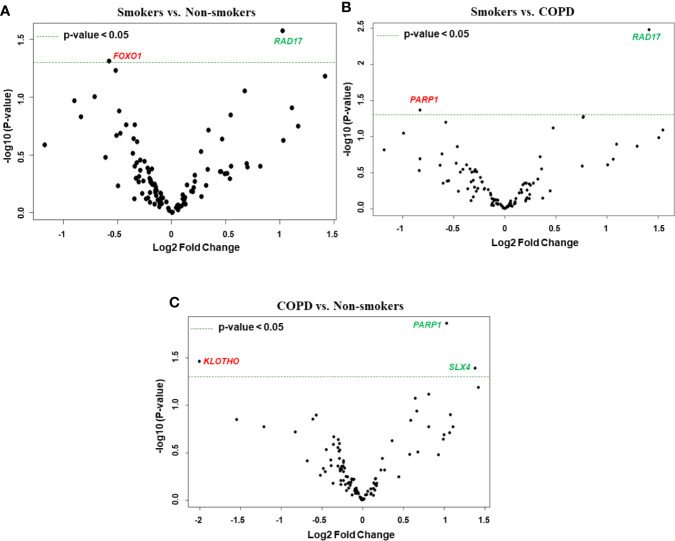
Volcano plots showing differentially expressed genes related to mitochondrial biogenesis and function, telomere replication and cellular senescence genes among non-smokers, smokers, and COPD subjects. Altered genes in comparisons between **(A)** smokers vs. non-smokers, **(B)** smokers vs. COPD, **(C)** COPD vs. non-smokers. The genes differentially expressed in each comparison are indicated in green (increased) and red (decreased) colors. The green dotted horizontal line indicates the significance threshold of p-values from comparisons at *P* < 0.05. The Benjamini-Hochberg procedure was further used to adjust the p-values to control the false discovery rate at 5%.

We performed grouped analysis of differentially expressed transcripts (comparisons without considering the age factor (young or old) among non-smokers, smokers and patients with COPD groups (e.g., young non-smokers with old non-smokers group, young smokers with old smokers group, and young COPD and old COPD group; n = 8/group; **Figures 6** and **7A**). Results indicated that smokers show decreased *FOXO1* and increased *RAD17* levels as compared to non-smokers group (**Figure 8A**). While decreased *PARP1* and increased *RAD17* levels were observed in smokers as compared to COPD group (**Figure 8B**). In patients with COPD *KLOTHO* was decreased and *PARP1* and *SLX4* genes were increased as compared to non-smokers group (**Figure 8C**). Furthermore, these comparisons revealed that smokers have significantly higher levels of *RAD17* expression compared to both non-smokers and COPD groups. Whereas, COPD patients showed significantly higher levels of *PARP1* as compared to both non-smokers and smokers groups.

### Altered Gene Expression Levels in Young and Old Non-Smokers Versus IPF Groups

Pairwise analysis of young non-smokers vs. young IPF showed 25 significantly altered genes, which were given in **Table 2**. Comparisons between old non-smokers and old IPF showed 13 significantly altered genes, as listed in **Table 3** along with their observed level of significance. The gene comparisons among these groups were given in **Figure 7B**.

**Table 2:** Altered genes in comparisons between young non-smokers and young IPF subjects.

List of genes that were found to be increased in young IPF compared to young non-smokers	
Genes	p values
AIFM2	0.027262
COX10	0.008096
IGF1	0.017368
RAD50	0.041025
**List of genes that were found to be decreased in young IPF compared to young non-smokers**	
AIP	0.007814
AKT1	0.028541
ATP5O	0.010191
CDK2	0.001304
CDKN1B	0.034287
ERCC1	0.043178
FIS1	0.016032
GADD45B	0.000329
GSK3B	0.000953
HNRNPD	0.035938
ID1	0.007673
IGF1R	0.017547
NFATC1	0.028756
NFATC2	0.015133
RAP1A	0.014367
RAPGEF1	0.025203
RELA	0.014566
SP1	0.024547
TGFB1	0.017524
TIMM10B	0.011584
UXT	0.034998

**Table 3:** Altered genes in comparisons between old non-smokers and old IPF subjects.

List of genes that were found to be increased in old IPF compared to old non-smokers	
Genes	p values
AIFM2	0.036937
ALDH1A3	0.004268
BAK1	0.002375
FEN1	0.017398
PARP1	0.029557
PCNA	0.001159
RPA2	0.022724
TERF2	0.013038
**List of genes that were found to be decreased in old IPF compared to old non-smokers**	
CDK2	0.016647
ERCC1	0.007071
FOXO1	0.04744
ID1	0.03526
IGF1R	0.014197

### Altered Gene Expression Levels in Young and Old COPD Versus IPF Groups

As detailed above both COPD and IPF are chronic age related diseases that severely alter lung function and share certain common features for the disease occurrence and progression. Here, we compared the altered gene levels related to the same pathways among COPD and IPF subjects as detailed above. There was no change in any of the genes analyzed in comparisons between young IPF (n = 3) and old IPF (n = 13). While, 16 genes were found to be altered in the comparisons between young COPD vs. young IPF, as indicated in **Table 4**. A total of 6 genes were altered in comparisons between old COPD vs. old IPF as shown in **Table 5**.

**Table 4:** Altered genes in comparisons between young COPD and young IPF subjects.

List of genes that were found to be increased in young IPF compared to young COPD	
Genes	p values
ALDH1A3	0.004086
COX10	0.019541
IGF1	0.024304
NOX4	0.001143
RAD50	0.014307
TNKS2	0.006571
UCP3	0.043174
**List of genes that were found to be decreased in young IPF compared to young COPD**	
AIP	0.041142
ATP5O	0.02557
CDK2	0.042857
ERCC1	0.039613
FIS1	0.011772
GADD45B	0.004059
ID1	0.009297
RHOT2	0.022329
SP1	0.013991

**Table 5:** Altered genes in comparisons between old COPD and old IPF subjects.

List of genes that were found to be decreased in old IPF compared to old COPD	
Genes	p values
ATP5L	0.033729
CDKN1B	0.026684
CDKN1C	0.010141
GADD45B	0.021874
SIRT2	0.018325
TSC2	0.033306

### Gene Expression Analysis of Differentially Expressed Targets

**Figure 9:**
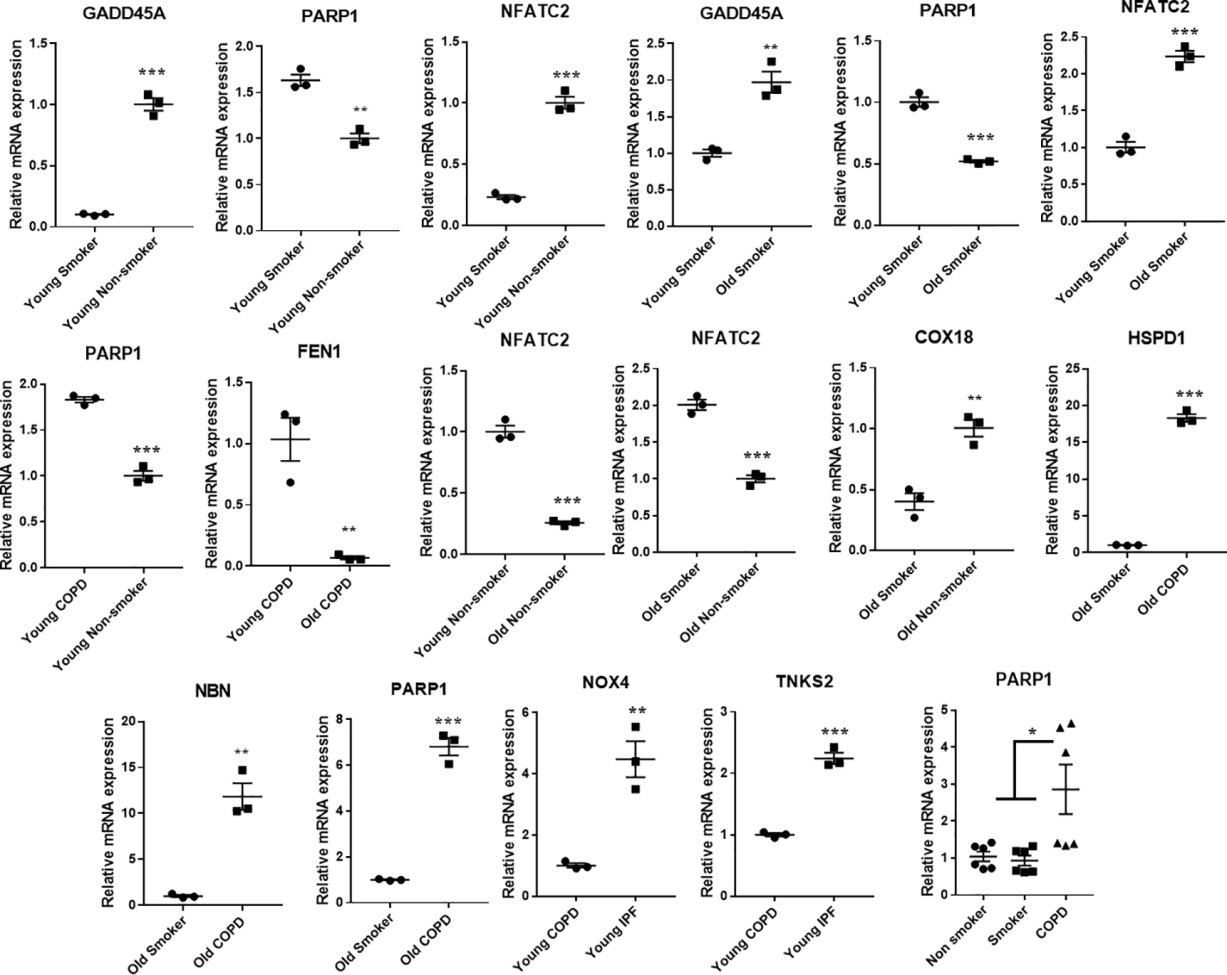
Quantitative PCR validation of the selected genes, which were found to significantly and differentially altered across various pairwise comparisons. The values were deduced based on 2-ΔΔCt method. The genes represented were found to be significant in their pairwise comparisons (p < 0.05). Student t-test was used to compare the level of significance in pairwise comparisons, while ANOVA was used for multiple comparisons.

Some of the differentially expressed mRNA targets predicted using Nanostring were selected for qPCR analysis. The expression trends of these selected genes matches with the trend observed using the NanoString mRNA analysis with varied levels of fold changes (**Figure 9**). Combined gene analysis for *PARP1* which matches with the trend as given in **Figure 8**. The results clearly indicate that the genes validated using qPCR are in agreement and significant across all the pairwise comparisons made.

### Protein Expression Levels of Crucial SARS-CoV-2 Targets in the Lung Homogenates

**Figure 10:**
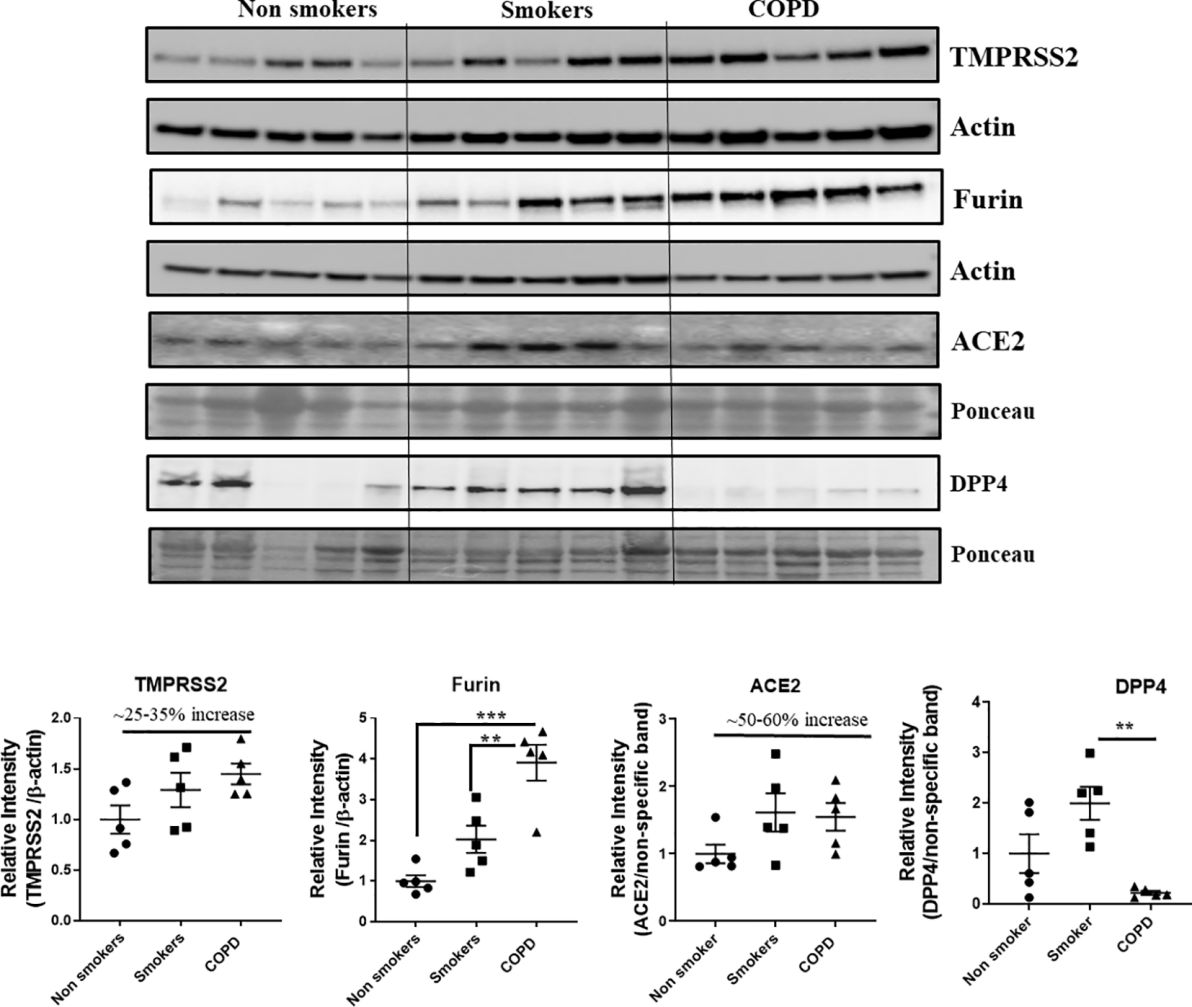
Western blot analysis of the crucial targets involved in COVID19 in non-smokers, smokers, and COPD subjects. Five samples per groups were used to probe for the TMPRSS2, furin, ACE2, and DPP4. Data were shown as mean ± SEM (n = 5/group). Level of significance were indicated as **P < 0.01 and ***P < 0.001 across the groups.

**Figure 11:**
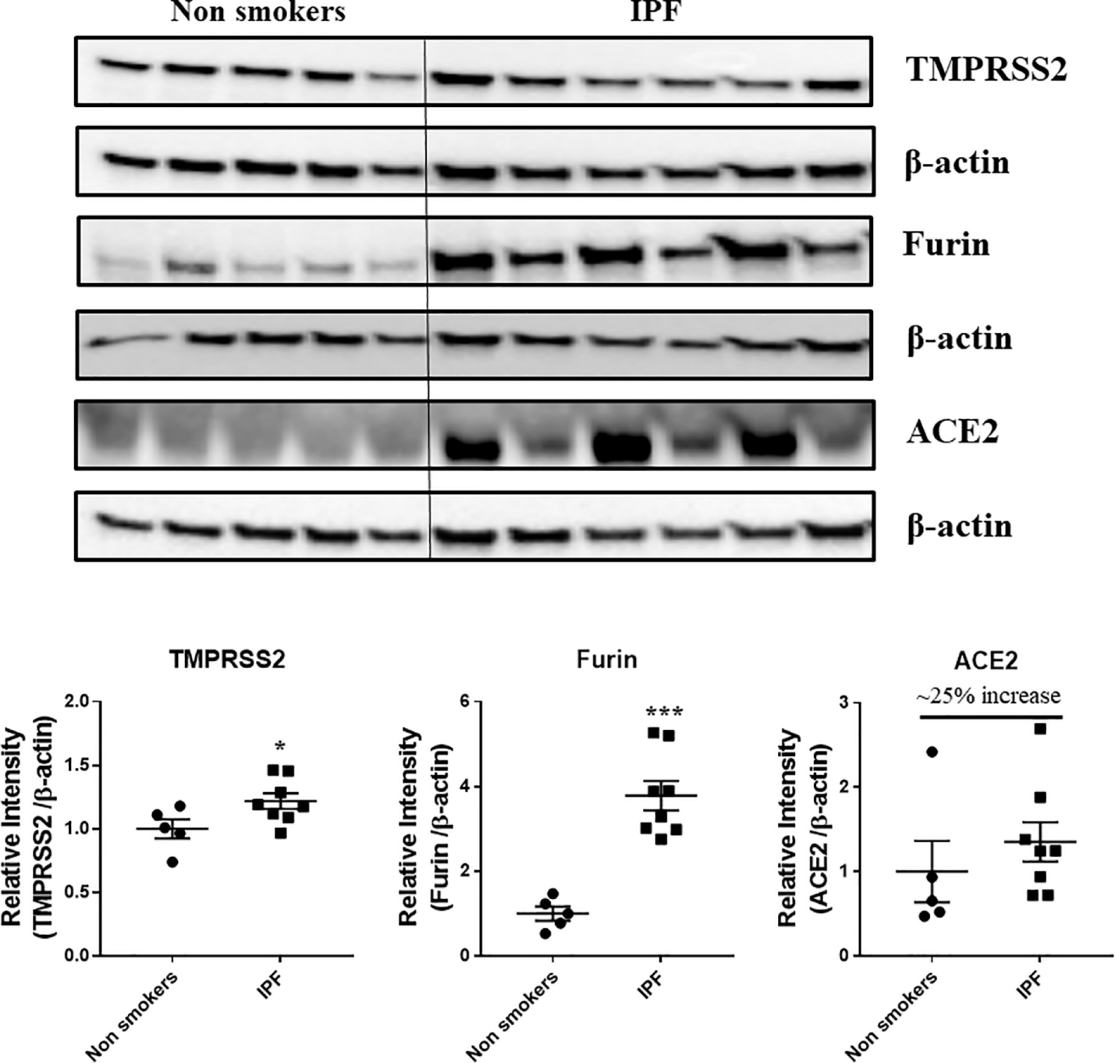
Western blot analysis of the crucial targets involved in COVID19 in non-smokers and IPF subjects. Five samples per groups were used to probe for the TMPRSS2, furin, and ACE2. Data were shown as mean ± SEM (n = 5/group). Level of significance were indicated as *P < 0.05 and ***P < 0.001 across the groups.

Western blots analysis (**Figures 10** and **11**, and **Supplementary Figures 1** and **2**) revealed a significant increase in the protein levels of TMPRSS2 protease (which plays a crucial role in the processing of the SARS-CoV-2 proteins) in COPD subjects as compared to smokers and non-smokers. Similarly, the levels of another important protease/convertase furin, which cleaves the spike protein of SARS-Cov-2, was also increased in smokers and COPD subjects, with a significant expression in COPD subjects. ACE2, cellular receptor for SARS-CoV-2 binding, was found to be increased in smokers as compared to the rest of the groups. The samples were also further probed for the expression of DPP4, another crucial protein which plays an important role in MERS‐CoV binding. The DPP4 expression was found to be significantly higher in smokers as compared to COPD and non-smokers. These results indicate that the levels of proteases, which aid in the processing and binding of the viral spike proteins were highly expressed in COPD and smokers. The expression results showed varied protein intensities in smokers and COPD for TMPRSS2 and DPP4 in the lungs, which may suggest a varied effect of virus entry/susceptibility based on specific cells in smokers and COPD/IPF subjects.

## Discussion

Aging is associated with the decline in lung function, and COPD is a disease of aging (Rojas et al., 2015; Wheaton et al., 2019). Nonetheless, there is growing evidence of COPD in younger subjects, which needs thorough and careful phenotypic characterization for potential markers to understand the cause and progression of disease. The current study examined the changes in gene expression in the lungs of non-smokers and smokers and patients with COPD/IPF. The study included age as an influencing factor independent of lung disease status. The extracted RNA was processed and analyzed using the sophisticated NanoString nCounter analysis platform. NanoString has several advantages in simultaneous estimation of the several gene levels in a single sample with low amounts of sample input (Eastel et al., 2019; Goytain and Ng, 2020). We have successfully used this platform to report changes in gene expression using different gene set panels in our earlier studies (Rashid et al., 2018; Sundar et al., 2018). In the current study, the custom designed panel included genes from three different pathways addressing the mitochondrial biogenesis and function, telomere function, and cellular senescence, which play a major role in the lung inflammation and COPD development.

Most chronic diseases, like COPD, are associated with the mitochondrial dysfunctions. Several reports claim the causal role of mitochondrial dysfunctions in the initiation and progression of smoking associated COPD (Bialas et al., 2016; Ryter et al., 2018; Zhang et al., 2020). We have previously reported the presence of mitochondrial dysfunctions in CS-induced lung damage models and in human lungs (Ahmad et al., 2015). Further, we along with others have reported that telomere dysfunction is also seen in COPD patients and smoking plays a crucial role in influencing telomere genes (Liu et al., 2002; Morla et al., 2006; Ahmad et al., 2017). Assessment of all these three pathway related genes in the same subjects throws light on the involvement and coordination of complex processes in smoking-related chronic lung disease such as COPD.

Pairwise comparisons revealed that a total of 21 genes were altered among the young and old subjects. *CDKN1A* (p21), a Cyclin–dependent kinase (CDK), plays a vital role in cellular senescence and proliferation and was reported to be increased in smokers and COPD subjects (Chiappara et al., 2013). Further, p21 disruption attenuated CS-induced lung inflammation in mice (Yao et al., 2008). In the current study, there was a reduced expression in the p21 levels in the young smokers compared to non-smokersin accordance with the previous reports, where *CDKN1C* (p57), along with p21 was decreased in aging lungs of mice (Park and Chung, 2001).

Among the other important targets *KLOTHO*, *SIRT6*, *PARP1*, and *PCNA* were altered in the current study. COPD patients has reduced KLOTHO expression as compared to non-smokers, in accordance with previous reports (Gao et al., 2015). In similar lines, *PARP1* was also elevated in the COPD subjects compared to smokers and non-smokers, as reported earlier (Hwang et al., 2010; Dharwal and Naura, 2018). We have reported that *TERT* levels were altered in mice (young and old) exposed to chronic CS (Rashid et al., 2018). Accordingly, the current results demonstrates that *TERT* levels were significantly increased in COPD patients, but this gene may be influenced in a different way in humans, as younger COPD patients has higher *TERT* levels compared to older ones. Nuclear factor of activated T cells c2 (*NFATC2*) levels were significantly higher in aged smokers as compared to younger ones. Earlier reports claim that *NFATC2* levels were increased by nicotine/smoking (Frazer-Abel et al., 2004). It was also reported that *NFATC2* enhances tumor-initiating phenotypes in lung adenocarcinoma (Xiao et al., 2017). These observations were opposite in non-smokers, as they age the levels of *NFATC2* decreased. This suggests that *NFATC2* may be used as a potential marker related to CS-induced lung damage. Among the other important genes that were altered and are crucial in the maintenance of these three pathways are ACD, which is related to *TPP1* gene and coordinates with its function was elevated in COPD (Else et al., 2007; Ahmad et al., 2017). *FEN1* could be a novel biomarker for COPD which was increased in the young COPD group as compared to the old COPD group. Prior studies showed mutation in *FEN1* linking lung cancer progression in an age-dependent manner in mice exposed to benzo[α]pyrene which is present in tobacco smoke (Wu et al., 2012; Ahmad et al., 2017).

Pairwise comparisons between non-smokers and IPF subjects revealed changes in some important genes like *PARP1*, *PCNA*, *FEN1*, *CDKN1B*, *NFATC2*, and *GADD45B*, as discussed in the above comparisons. However, the directionality of the changes varied between groups, which may be attributed to the small sample size in the study and the heterogeneity in the samples used. Further comparisons were also made between the expression profiles of the young IPF and old IPF with their age matched COPD subjects. Interestingly, some of the well characterized genes in IPF like *NOX4* and *TNKS2* were increased in the young IPF as compared to the young COPD patients (Hecker et al., 2014). Mitophagy is a well-known phenomenon occurring in both COPD and IPF and regulates the mitochondrial related damage response toward the disease (Hara et al., 2018; Ryter et al., 2018). Genes participating in the mitochondrial dynamics and other quality control mechanisms like *FIS1* and *RHOT2* were found to be decreased in young IPF compared to their age matched COPD subjects. *ERCC1* (Excision Repair Cross-Complementation Group 1) was also found to be high in young COPD as compared to IPF. Earlier reports claim that *ERCC1* gene is strongly associated with COPD subjects (de Andrade et al., 2012). Some of the common gene targets like *GADD45B* needs further attention and characterization especially in the chronic lung diseases like COPD and IPF. Recent biomarker identification study indicated *GADD45B* in their list of genes that can be targeted in chronic diseases like asthma, IPF, and COPD (Maghsoudloo et al., 2020).

Several studies indicate that the patients with underlying chronic disease conditions like diabetes, hypertension, and COPD are more prone to COVID-19 infections and have higher chances of the hospitalization rates and mortality (Emami et al., 2020; Koff and Williams, 2020; Zhao et al., 2020). Several crucial mechanisms and targets were reported to increase this susceptibility in these patients (Hoffmann et al., 2020). In the current study, we have determined the expression of four such important protein targets reported to play an important role in SARS-CoV-2 COVID-19. As anticipated, COPD and IPF patients in our study have higher levels of TMPRSS2 proteins in the lungs, suggesting the ideal condition for the processing of the viral protein and attachment to its receptor ACE2. ACE2 receptor abundance expression was slightly increased in smokers, COPD, and IPF subjects (Leung et al., 2020). Furin, another crucial protease in COVID-19 infection, was also found to be higher in smokers, COPD, and IPF as compared to the non-smokers. We have also assessed the levels of DPP4 in the same samples and found that DPP4 was significantly higher in the smokers as compared to non-smokers and COPD. DPP4 was found to play an important role in the entry of MERS-CoV, acts as its receptor (belonging to the similar class of beta coronaviruses) in humans, and was also suggested to play an important role in COVID-19 infections (Bassendine et al., 2020). In agreement with recent reports (Seys et al., 2018), suggesting that smokers and COPD have higher DPP4, our findings suggest increase in DPP4 in smokers, but to our surprise we didn’t find any increase in COPD subjects. This may suggest a different mechanism in smokers and COPD, which required further studies. Nevertheless, the varied abundance of different SARS-CoV-2 COVID-19 proteins suggest cell-specific effects for viral entry in smokers and COPD/IPF subjects. This may be used as pharmacological targets in attenuating COVID-19 infections.

In conclusion, our study provides a novel direction showing a crucial and interdependent association with different cellular pathways, e.g., mitochondrial, telomere, and cellular senescence in association with SARS-CoV-2 COVID-19 proteins. Whilst the study provides several differential gene expression patterns in non-smokers, smokers, and COPD/IPF, there is a limitation regarding the small sample size and past smoking history in terms of their stratifications for further analyzing the data and their interpretations. It remains to be seen the alterations seen in various genes will have direct bearing on susceptibility to COVID-19 infections in smokers, and patients with COPD and IPF. Nonetheless, this human study provides useful and valuable information in terms of approach and identifying targets involved in various cellular processes linking with age and COPD and IPF disease progression with pharmacological targets in COVID-19 infections.

## Data Availability Statement

The raw data supporting the conclusions of this article will be made available by the authors, without undue reservation, to any qualified researcher.

## Ethics Statement

The current study was approved for the procurement of the human lung tissues as de-identified tissues by the Materials Transfer Agreement and Procurement (Institutional Review Board, IRBC), and laboratory protocols by the Institutional Biosafety Committee, IBC of the University of Rochester Medical Center, Rochester, NY, with Project Code: DRAI1 001 Protocol: 004, Date of approval and IRB/IBC approvals 2/11/2017 and 2/7/2018, and 9/29/2017 and 2/10/2017, University material transfer agreement (MTA) agreements signed on the above dates as well. Written informed consent was not provided because The human peripheral lung tissues from non-smokers, smokers, and COPD/IPF were procured/obtained from the NDRI (National Disease Research Interchange) and LTRC [Lung Tissue Research Consortium of the National Heart Lung Blood Institute (NHLBI)].

## Author Contributions

KM: Experiments, data analyses, writing, and editing the manuscript. IS: Data analyses and editing the manuscript. DL: Data analyses and editing the manuscript. IR: Concept, design, obtained funding, writing, and editing the manuscript.

## Funding

This study was supported by the NIH R01 HL1377380, R01 HL135613, and R01 ES 029177 (all IR). DL is supported in part by the University of Rochester CTSA UL1 TR002001 of the NIH.

## Conflict of Interest

The authors declare that the research was conducted in the absence of any commercial or financial relationships that could be construed as a potential conflict of interest.

## Supplementary Material

Supplementary Table 1 Pre-selected genes belonging to the mitochondrial biogenesis and function pathway with their annotations.

Supplementary Table 2 Determined genes with raw and normalized counts based on Nano String analysis for non-smokers, smokers, and COPD comparisons.

Supplementary Table 3 Determined genes with raw and normalized counts based on Nano String analysis for non-smokers, smokers, COPD and IPF comparisons.

Supplementary Figure 1 and 2 Full unedited gels/blots for **Figures 10** and **11** (original and unprocessed) are shown.
