# Supplementary material for: Age-Dependent Assessment of Genes Involved in Cellular Senescence, Telomere, and Mitochondrial Pathways in Human Lung Tissue of Smokers, COPD, and IPF: Associations With SARS-CoV-2 COVID-19 ACE2-TMPRSS2-Furin-DPP4 Axis
Source: Front Pharmacol. 2020 Sep 9;11:584637. doi: 10.3389/fphar.2020.584637 (PMC7510459; doi:10.3389/fphar.2020.584637)

Supplementary Figure 1. **Full unedited gels/blots for Fig. 10 (original and unprocessed)**

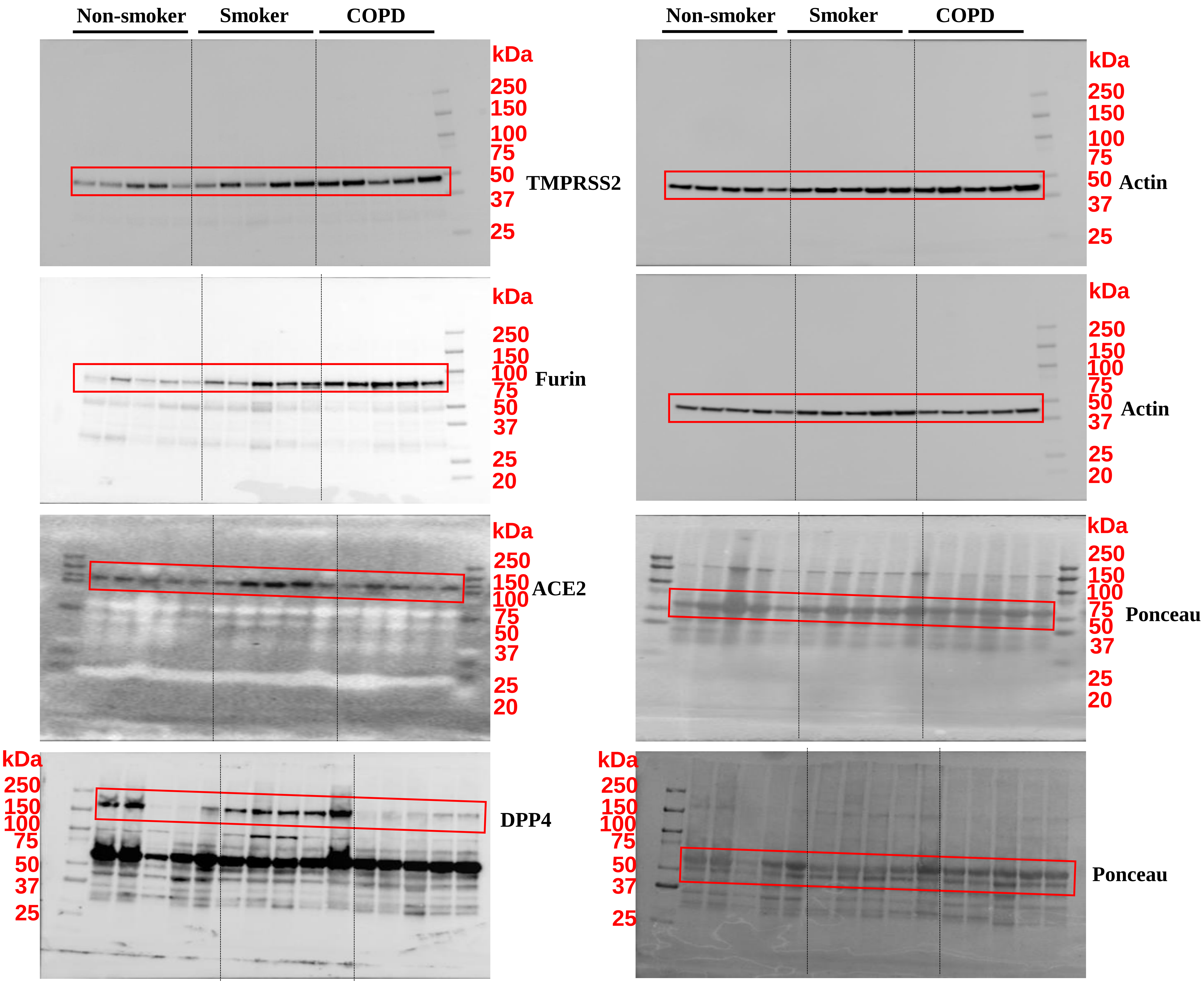

Supplementary 2.

Full unedited gels/blots for Fig. 11 (original and unprocessed)

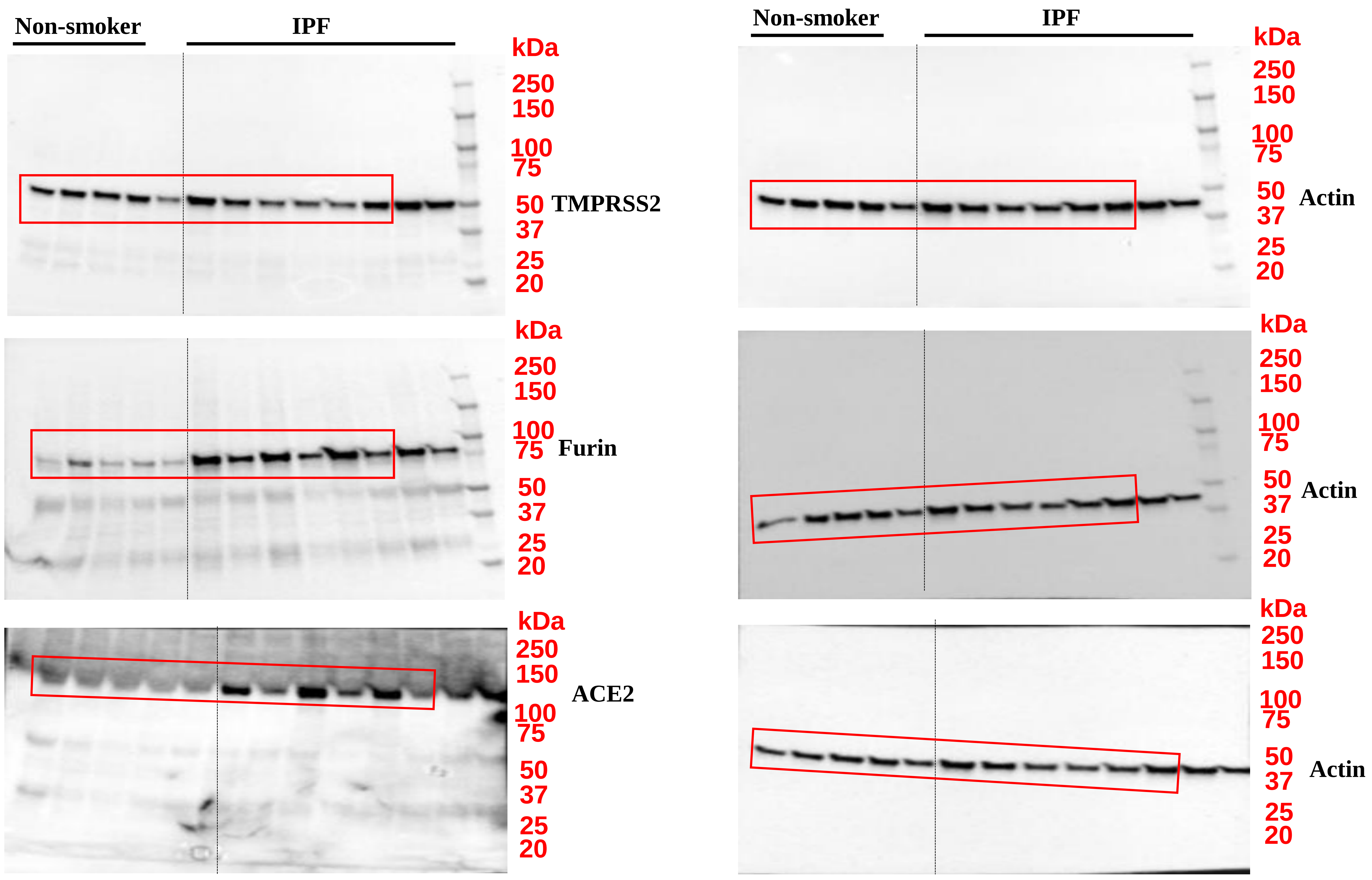

Supplement: Supplementary Figure 1 and 2 — Full unedited gels/blots for Figures 10 and 11 (original and unprocessed) are shown. [file DataSheet_1.pdf]
